# Volume Segmentation and Analysis of Biological Materials Using SuRVoS (Super-region Volume Segmentation) Workbench

**DOI:** 10.3791/56162

**Published:** 2017-08-23

**Authors:** Michele C. Darrow, Imanol Luengo, Mark Basham, Matthew C. Spink, Sarah Irvine, Andrew P. French, Alun W. Ashton, Elizabeth M.H. Duke

**Affiliations:** ^1^Science Division, Harwell Science and Innovation Campus, Diamond Light Source; ^2^School of Computer Science, University of Nottingham

**Keywords:** Basic Protocol, Issue 126, Segmentation, Supervoxels, Cryo Electron Tomography, Cryo Soft X-ray Tomography, Phase Contrast X-ray Tomography, Machine Learning, SuRVoS Workbench

## Abstract

Segmentation is the process of isolating specific regions or objects within an imaged volume, so that further study can be undertaken on these areas of interest. When considering the analysis of complex biological systems, the segmentation of three-dimensional image data is a time consuming and labor intensive step. With the increased availability of many imaging modalities and with automated data collection schemes, this poses an increased challenge for the modern experimental biologist to move from data to knowledge. This publication describes the use of SuRVoS Workbench, a program designed to address these issues by providing methods to semi-automatically segment complex biological volumetric data. Three datasets of differing magnification and imaging modalities are presented here, each highlighting different strategies of segmenting with SuRVoS. Phase contrast X-ray tomography (microCT) of the fruiting body of a plant is used to demonstrate segmentation using model training, cryo electron tomography (cryoET) of human platelets is used to demonstrate segmentation using super- and megavoxels, and cryo soft X-ray tomography (cryoSXT) of a mammalian cell line is used to demonstrate the label splitting tools. Strategies and parameters for each datatype are also presented. By blending a selection of semi-automatic processes into a single interactive tool, SuRVoS provides several benefits. Overall time to segment volumetric data is reduced by a factor of five when compared to manual segmentation, a mainstay in many image processing fields. This is a significant savings when full manual segmentation can take weeks of effort. Additionally, subjectivity is addressed through the use of computationally identified boundaries, and splitting complex collections of objects by their calculated properties rather than on a case-by-case basis.

**Figure:**
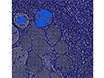


## Introduction

The SuRVoS Workbench is a piece of software designed to allow researchers to extract scientifically relevant information from volumetric data from various samples, regardless of the structure of interest, resolution or the imaging modality^1^,^2^. Volumetric data such as these are often collected using X-ray or electron tomography systems, routinely based at large laboratories or centralized facilities due to their complexity. Both of these methods, and other techniques, produce large, information rich datasets which prove challenging to segment with either semi-automatic methods or manually. Particularly, near-native state cryo-immobilized datasets require low-dose imaging conditions, resulting in a low signal-to-noise ratio and poor contrast, especially in cryo electron tomography (cryoET)^3^,^4^,^5^. An additional factor in some 3D datasets is the presence of artifacts introduced by the challenging experimental conditions involved, for example missing wedge artifacts due to data collection over a limited tilt range, resulting in missing information and elongation in the direction of the beam^3^,^4^,^5^. Even when low signal-to-noise or missing wedge artifacts are not problematic (e.g. focused ion beam SEM^6^ or serial block face SEM^7^), the complexity and three-dimensional nature of the sample, and the large amount of data mean analysis would still benefit from an automated process for data segmentation.

Currently, when considering biological volumes of cells, there are many options for automatically or semi-automatically identifying very specific cellular features, such as actin, microtubules, or specific protein complexes, using a template-based search, or identifying features in specific types of datasets (*e.g.* high contrast, stained, resin-embedded samples)^8^,^9^,^10^,^11^,^12^. However, in these cases *a priori* information or specific sample preparation protocols are necessary, limiting the broad applicability of these segmentation strategies. There are also tools available that perform model training at the voxel level to learn the appearance of various structures of interest when given user input^13^. However, at this level the complexity of training and testing the models can be error-prone and computationally expensive. Given the challenging image conditions, and the lack of broadly applicable, semi-automatic segmentation strategies, manual segmentation is common, even when working with complex biological materials^14^,^15^,^16^,^17^. However, it is generally accepted that the process of manual segmentation is not only time-consuming, but also error-prone, subjective and variable^4^,^5^,^18^,^19^,^20^. Some segmentation programs offer tools to ease the manual segmentation process (*i.e.* interpolation, lasso, or blow tools)^21^,^22^, however, in cases of noisy datasets, they are difficult to apply successfully, and even when they are used successfully, the process is still subjective and variable.

Traditionally, segmentations have been used in two distinct ways: qualitatively or quantitatively. As imaging technologies and segmentation strategies improve, it has become more common to use segmentation as a quantitative tool to answer biological questions and as a "ground truth" for algorithm development^8^,^12^,^15^,^23^,^24^,^25^. In order to do this, detailed checks and balances are required to decrease variability and subjectivity throughout the process^26^. However, these precautions further increase the time-consuming nature of segmentation. Because of this, it is critical to provide a faster and less variable segmentation strategy.

The SuRVoS Workbench begins to address these issues by providing the user with a selection of machine learning and image processing tools that assist the user in the segmentation process, while also guiding the user through the required steps. To achieve this, two key innovations are implemented together in SuRVoS. First, it uses a super-region hierarchy to group similar, nearby regions of the data based on their inherent properties. Each of the regions in the hierarchy represents the same volume using fewer elements, while still providing strong boundary adherence. Thus, super-regions reduce the complexity of segmenting a volume by several orders of magnitude yet still represent the data without significant loss of information^27^. Second, SuRVoS provides a semi-automated segmentation strategy that uses minimal manual segmentation inputs to train classifiers, which are then used to segment the remaining volume^28^,^29^. This strategy reduces manual segmentation, greatly decreasing the amount of user time spent on segmenting and, when using super-regions, removes manual delineation of boundaries, potentially reducing variability and subjectivity.

A further key feature of SuRVoS is the Label Splitter Tool, whereby a user can classify a series of already segmented objects based on their inherent properties. After segmentation of various objects of interest, this tool can be used to divide the set into subclasses based on measures such as average object intensity, variance, size, location, *etc.* This is useful when classifying large groups of objects with high complexity. For example, a group of cellular organelles can be split into mitochondria, empty vesicles, lipid droplets, etc.; or a set of material inclusions can be separated based on size or shape. Once segmented the individual labels can be split into groups using any number of classifiers, reducing identification bias.

The SuRVoS Workbench has been successfully used to segment data from several imaging techniques. Here, synchrotron X-ray phase contrast tomography (microCT) of the fruiting body of a plant is used to demonstrate segmentation using model training, cryo electron tomography (cryoET) of human platelets is used to demonstrate segmentation using super- and megavoxels, and cryo soft X-ray tomography (cryoSXT) of a mammalian cell line is used to demonstrate the label splitting tools

## Protocol

NOTE: Generally useful ranges of parameters for each processing step and the specific parameters for each data type shown here are provided in **Table 1**.

### 1. Preparing a Workspace and Loading Data

1. Launch SuRVoS Workbench, click the open dataset button, and in the resulting popup, select the data file to be segmented. Choose an appropriate orientation of the dataset. Next, choose or create a folder where the workspace and associated files will be stored. It is recommended that this folder is empty when starting a new segmentation. After the data has been loaded, the workbench will open with the Plugins pane on the left, the Visualization pane on the right and a set of Tool shortcuts between the two panes (Figure 1).

### 2. Preprocessing and Data Representation

1. In the Select ROI tab, input the z, y, and x start and end coordinates for the region of interest and click add. To choose appropriate y, and x coordinates hover the mouse over a point on the image. Choose z coordinates using the slider at the top of the Visualization pane. Once a region has been added, make sure it is selected by checking the box to the right. All downstream calculations will be performed on the selected region. Generally, starting with a small, representative region of interest (ROI), optimizing parameters and then re-applying these parameters to the whole area to be segmented is advised.
2. In the Feature Channels tab, use the drop-down menu at the top to choose a feature/filter and add it to the queue (see Discussion for more info about feature channels). Once a feature/filter has been added and selected by clicking on its name, modify options specific to the feature/filter and choose the input dataset on which to run the feature/filter. Once all options have been chosen, click the checkbox to the right of the feature/filter name to compute.
  1. In order to optimize parameters for a new dataset, add multiple filters/features and choose the parameters for them, before being computed in order, one after the other. To do this, add each new filter/feature and select appropriate parameters, check the box to the left of each filter/feature to be run, and click the compute features box at the top of the pane. See Discussion for additional information.

### 3. Generating Appropriate Super-Regions

1. In the Super Regions tab, in the Supervoxels section, use the source drop-down menu to select the filtered dataset from which supervoxels will be created. Then specify the shape, spacing and compactness of the supervoxels (see Discussion and Figure 2 for further details). Finally, click on the apply button to generate the supervoxels. Once the supervoxels have been created they can be viewed in the Visualization pane, turned on or off and their transparency controlled in the Visualization tab and Viewer Window shortcut.
2. In the Super Regions tab, in the Megavoxels section, use the source drop-down menu to select the filtered dataset from which megavoxels will be created. Next, specify the lambda, numBins and Gamma parameters of the megavoxels (see Discussion for further details). Once the megavoxels have been created they can be viewed in the Visualization pane, turned on or off and their transparency controlled in the Visualization tab and Viewer Window shortcut.

### 4. Introduction to Annotation

1. In the Annotations tab use the Add Level button to add an annotation level. After a Level has been added, use the Add Label button in that level to add a label for the annotation. Once added, the name and color of the label can be modified for ease of annotation.
2. Next, in order to begin annotating, select the pen icon from the tool shortcut section. When this is selected a set of options appear at the top of the Visualization pane. These options control the pen width and whether voxels, supervoxels or megavoxels will be used to annotate.
  1. For the purpose of model training, generally, select supervoxels in the Annotation Level drop-down box and a middling to large pen width should be used. In the annotations tab, select the label to be annotated by checking the box to the far right of the label information. Next, click in the Visualization pane to annotate a single supervoxel, or click and drag to annotate many. NOTE: Voxels and megavoxels can be selected in the Annotation Level drop-down box and used to annotate in the same way, which, in the case of megavoxels, can enable many thousands of similar voxels to be segmented with a single click of the mouse.

### 5. Segmentation Using Model Training demonstrated with a microCT dataset.

NOTE: The first segmentation for many datasets is to differentiate multiple large regions from each other. For example, separating the nucleus from the cytoplasm, or the cell from the external ice and support structure. For this type of segmentation, with clear delineated boundaries and large regions, model training is useful. To demonstrate this, X-ray phase contrast tomographic data of Goosegrass will be used.

1. Load the data, preprocess using the filter and feature suite, and determine appropriate supervoxels and/or megavoxels as described in the above sections using the parameters in **Table 1** as a guide. Continuing to use the parameters from **Table 1 **and the instructions in Section 4, roughly annotate some large areas of the dataset, as shown in Figure 3. NOTE: The dataset does not need to be completely segmented at this point.
2. In the Model Training tab, set the predict level to the level that contains the manual training annotations, and in the descriptor section set the region to Supervoxels. Next, select the descriptors that are to be used to differentiate regions of the data by clicking on the Select Sources drop-down and checking the boxes of the features and filters of choice (see **Table 1** and discussion).
3. Next, click on the predict button. After computation is complete, the Visualization pane will be updated with the predictions for all of the non-labeled voxels showing which of the annotation classes they are predicted to belong to. Generally, the default parameters for each classifier methodology provide a good starting point and the user should only need to switch between classifiers to find a good fit. However, for expert or experienced users the options for each classifier are available and can be modified.
4. After assessing the effect of the training methodologies and choosing one, apply additional refinement by clicking on the Refine drop-down in the Refinement section. At the bottom of the Model Training tab, in the "Update Annotations" section, make sure that the visualization drop-down menu is set to Predictions. Use the confidence slider to assign more or less of the unannotated supervoxels to the selected annotation labels.
5. After an appropriate level of confidence has been selected based on visual inspection, use the Save buttons next to the labels at the bottom of the Confidence tool to save the predictions into specific labels. The Visualization pane will update to show the changes. Each label can be saved separately, and indeed, labels can be saved from smaller sub-regions by inputting values in the From and To z, y, and x boxes and clicking on the Save button for each label.
6. Address minor mislabeling by providing further training data as described in Section 4. After appropriate predictions are added to labels, repeat the process of model training with refinement and adding high confidence predictions until there are no more unlabeled supervoxels. This is effective because each time the model training process is run there are more assigned supervoxels to train with, and hence the process becomes more robust as the iterations increase.

### 6. Segmentation using Super-Regions, Demonstrated with a CryoET Dataset.

NOTE: Since Super-Region segmentation is useful for smaller, discretely bound areas, the focus here will be on segmentation of the organelles and microtubules within this dataset. Model training was used to quickly segment the platelet from the background ice and carbon; these parameters are not discussed further, but are presented in **Table 1**.

1. Load the data, preprocess using the filter and feature suite, and determine appropriate supervoxels and/or megavoxels as described in the above sections using the parameters in **Table 1** as a guide.
2. Add appropriate levels and labels to the annotation tab, select a label and begin annotating using a middling pen width with supervoxels selected. Be mindful of the need to choose different labels for objects in close proximity to each other in order to avoid labeling them as a single object.
3. In order to clean up the annotations further, use morphological refinement methods (dilation, erosion, opening, closing and fill holes). These options can be found at the bottom of the Annotations tab. To use them, select the segmentation label and refinement method. Enter a radius value and choose how to apply the refinement. Then click refine.

### 7. Classification and Analysis of Data Objects Based on Inherent Characteristics, demonstrated with a CryoSXT Dataset

NOTE: Generally, the next step after segmentation is analysis of the data. The label splitter tool in SuRVoS allows for the classification of segmented objects using rules based on intrinsic characteristics of the objects such as average object intensity, variance, volume, or position. The label statistics tool allows for the visualization of relationships between these measures for each new object class. These are powerful new tools for the analysis of complex 3D datasets after segmentation.

1. Load the data, preprocess using the filter and feature suite, determine appropriate supervoxels and/or megavoxels and segment as described in the above sections using the parameters in **Table 1** as a guide.
2. After segmentation, click on the second tab of the Visualization pane, called Label Splitter. This will add a new area to the right-hand side of the window - the Rule Creation pane.
3. At the top of this area, select an appropriate level and labels for label splitting. Then select the dataset to query and click Label. All of the objects in the selected labels will now be outlined in blue as separate objects in the Visualization pane and a plot showing the average intensity of the objects will be displayed in the Rule Creation pane. To change the measure being shown in the plot, click on the drop-down box at the top of the right-hand side.
4. To begin to split the objects into relevant classes, click add new label at the bottom of the Rule Creation pane. The name and color associated with this new label can be changed as described previously.
  1. Click Add new rule and using the drop-down and freeform entry boxes define the rule to be applied. Click Apply to see the effects of the new rule in the Visualization pane and the plot in the Rule Creation pane. Multiple rules can be applied to a single label and multiple labels can be created within the same dataset. NOTE: In order to gather any unlabeled objects, create a new label and instead of adding a rule to it, click Select Others.
5. When the objects of interest have been split into new labels, create a new, empty level in the Annotations tab. Then choose this level in the Rule Creation Tab and click Save labels. This will save the new labels to this empty level.
6. On the edge of the Visualization pane, click on the Label Statistics tab. This will open a new Visualization pane that can be used to begin to understand relationships between object classes in the data. At the top, select an appropriate level and labels, and the dataset to query.
  1. Select a few of the measures of interest by checking the boxes next to them. Then click Label. This will produce pairwise comparison plots for each of the selected measures. To update the plots, select or deselect appropriate measures then click Update plot.

### 8. Exporting Data and Segmentations

1. Export the plots and the raw measurement data by clicking on Export plot and Export data, respectively, in the Label Statistics tab of the Visualization pane.
2. Export the image data and segmentations by clicking on the Export tab in the Plugins pane. First, click to select a folder where the data will be saved. Next, select the output (Raw Data, Raw Annotations, Segmentation Masks, or Masked Data) and the format (HDF5, MRC, or TIFF). Lastly, select the Annotation level(s) to be exported using the check boxes and click Export. The data can be scaled and inverted prior to exporting as necessary. When exporting the masked data, the dataset that the mask(s) will be applied to can be selected using a drop-down menu.

## Representative Results

Three volume datasets collected from three different techniques (microCT, cryoET, and cryoSXT) were used to demonstrate three important features of SuRVoS Workbench: Model training, super-region segmentation and label splitting. The datasets represent a diverse group of experimental results, for each of which full processing parameters are provided (**Table 1**).

To demonstrate model training using SuRVoS Workbench, a relatively high-contrast dataset with region defining boundaries was chosen. This dataset of the fruit of *Galium aparine*, or goosegrass, was collected using X-ray phase contrast tomography on the I13-2 Diamond-Manchester Imaging Beamline at Diamond Light Source, Chilton, Oxfordshire, UK. The fresh sample was mounted in air onto a goniometer base on top of the rotation stage, at a sample-detector distance of 30 mm. Exposure times were 0.10 s with the pink beam spectrum which has a mean energy of around 22 keV. Projections were collected through 180° with a step size of 0.1°. Tomographic reconstructions were performed using Savu^30^,^31^ with the Paganin filter for propagation-based phase contrast images^32^ followed by filtered back projection reconstruction in the ASTRA toolkit^33^,^34^. This data was then downsized with 2 x 2 x 2 binning to reduce the file size before being input into the SuRVoS Workbench.

First, the input data (Figure 3A) was filtered and clamped (to remove upper and lower intensity values in the data) (Figure 3B). In this way, the background and foreground were made more easily distinguishable and the texture of the internal structure of the fruit was accentuated. Next, supervoxels were built on top of the filtered dataset (Figure 3C). To assess the quality of the supervoxels, they were displayed without the data to verify that pertinent details of the dataset were well represented by the supervoxels (Figure 3D). Next, manual annotations using supervoxels were provided as training data on three slices of the volume (Figure 3E, dark colors). This training data was sufficient to train the classifier to predict (light colors) the areas corresponding to the background (green), fruit bristle (red), seed material (purple), and surrounding flesh (blue). Morphological refinements were used to clean up the segmentations by filling holes, growing or shrinking as needed (Figure 3F). The total time spent to determine appropriate parameters and segment this dataset was 2 h.

To demonstrate super-region segmentation using SuRVoS Workbench, a noisy and complex dataset was chosen^15^. This dataset was collected using cryoET at the National Center for Macromolecular Imaging at Baylor College of Medicine, Houston, TX USA. Briefly, platelets were plunge frozen on glow discharged and gold fiducial-treated holey carbon TEM grids. Tilt series were collected from ±65° with a 2° increment. The tilt series was then reconstructed using weighted back projection in IMOD^35^.

After loading the data into SuRVoS (Figure 4A), a region of interest was selected and an appropriate filter set was applied. In this case, a smoothing Gaussian filter followed by a total variation filter with the contrast clamped was used to accentuate the edges and textures of the data (Figure 4B). Next, model training with minimal supervoxel-based user input was used to segment the platelet from the background ice and carbon. Then, semi-manual segmentation with megavoxels and supervoxels was used to segment the organelles. Lastly, the supervoxel source parameter was changed to a weaker denoising filter and supervoxel shape was made smaller (see **Table 1**) in order to better preserve the microtubules boundaries for segmentation (Figure 4C). For both the organelles and the microtubules, quick manual annotations were used every 5 - 10 slices to select the supervoxels that describe the feature of interest (Figure 4D & 4E). The total time spent to determine appropriate parameters and segment the region of interest presented was 6 h.

To demonstrate label splitting using SuRVoS Workbench, a dataset with many, varied organelles was chosen. This dataset was collected using cryoSXT on beamline B24 at Diamond Light Source, Chilton, Oxfordshire, UK^36^. Briefly, HEK293 cells were grown on gold finder grids, appropriately sized gold fiducials were added and the grid was plunge frozen using a EM with back-sided blotting. Tilt series were then collected on a microscope from ±65° with a 0.5° increment. The tilt series was then reconstructed using weighted back projection in IMOD^35^.

After loading the data into SuRVoS (Figure 5A), a region of interest was selected and an appropriate total variation filter was used to enhance the boundaries of the organelles throughout the volume (Figure 5B). Next, organelles were semi-manually segmented using megavoxels and supervoxels, and then refined with fill holes, closing and dilation to smooth edges (Figure 5C). The total time to determine appropriate parameters and segment the region of interest presented was 4 h. Once the segmentation was finalized, the Label Splitter was used to visualize each organelle as an object in the dataset (Figure 5D) and various characteristics about each object in the data plot (Figure 5E). The Label Splitter interface is interactive, updating the color associated with each new label class in both the visualization and the data plot. This allows for the creation of various rules based on the characteristics inherent in the data that can be used to separate the objects into useful classes (Figure 5F).

**Figure:**
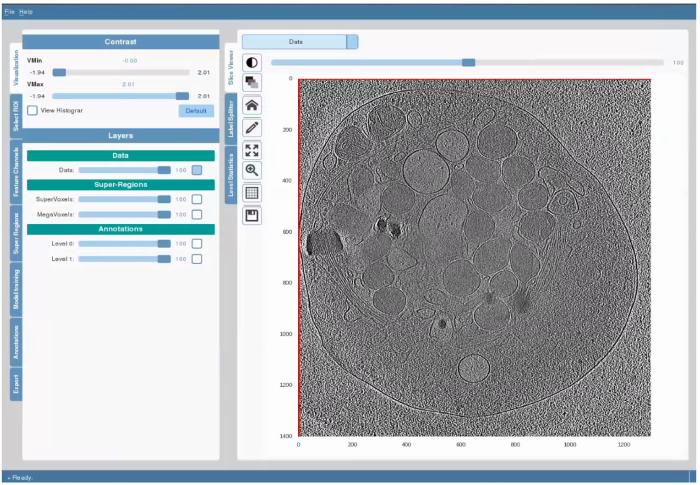

**Figure 1. The Layout and General Features of SuRVoS Workbench.** The GUI interface is on the left, while the Visualization pane is on the right. These two areas are separated by a column of tools and shortcuts. The GUI is arranged to walk the user through the main steps in preprocessing the data, choosing supervoxel and/or megavoxel parameters, segmenting the data, and if needed using model training, prior to exporting the segmentations. The Visualization pane can be used in three modes: basic visualization and segmentation to view the data and any applied filters and to segment the data, label splitter to categorize objects into new labels based on aspects inherent to the data, and finally label statistics to measure and visualize characteristics of the segmented objects. For each of these modes, the drop-down menu in the top left corner controls which data is shown, and the slider at the top controls the z-axis. The Tool shortcuts provides easy access control over contrast, transparency of layers, zooming, panning and returning to "home" in the Visualization pane, and opening tools for annotation as described in protocol. Please click here to view a larger version of this figure.

**Figure:**
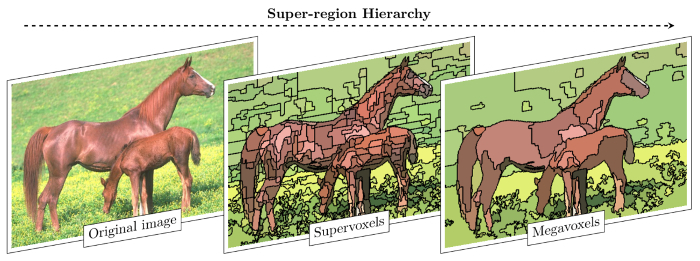

**Figure 2. Super-region Hierarchy Reduces Complexity of Image Segmentation.** An image from the Berkeley Segmentation Dataset (BSDS500^37^) was used to demonstrate the properties and effects of super-regions. The original image (left) is composed of thousands of voxels, which are then gathered into adjacent, similar groupings to create a few hundred supervoxels (center). Supervoxels can also be gathered into adjacent, similar groupings to create a few tens of megavoxels (right). With each grouping, the complexity of the segmentation task is decreased, for both computational and manual resources. Importantly, a 2D example is shown here, however, both supervoxels and megavoxels are 3D. Please click here to view a larger version of this figure.

**Figure:**
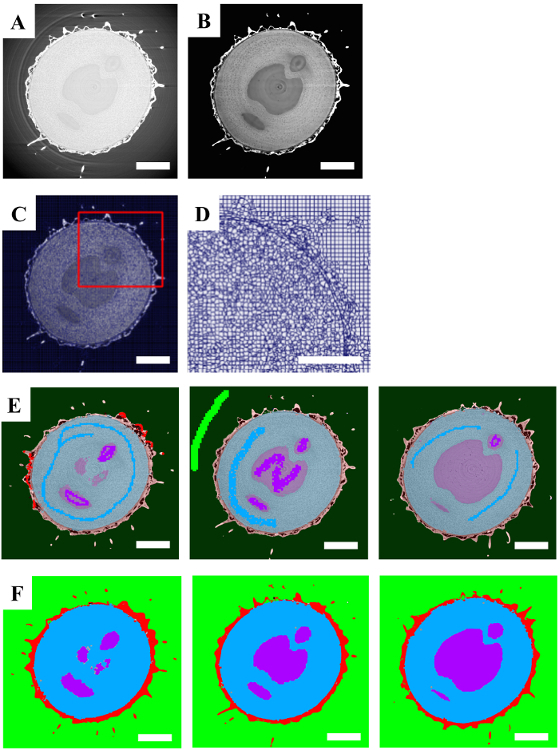

**Figure 3. Processing of a microCT Dataset using a Model Training Segmentation Strategy. A.** A single 2D slice of the raw data. **B.** Applying a clamped total variation filter to the raw data enhanced the boundaries between the various aspects of the fruiting body. **C.** Appropriate supervoxel parameters were chosen. **D.** A region of interest (red box in **C**) is shown to demonstrate that the boundaries of the data are present in the supervoxels themselves. **E.** Three slices of the volume with manual annotations of various areas of the dataset displayed in dark colors (green, red, blue and purple) and predictions after running model training displayed in the same light colors. **F.** The same three slices with the final segmentation after the model training predictions have been accepted. Scale bars are 1 mm. Please click here to view a larger version of this figure.

**Figure:**
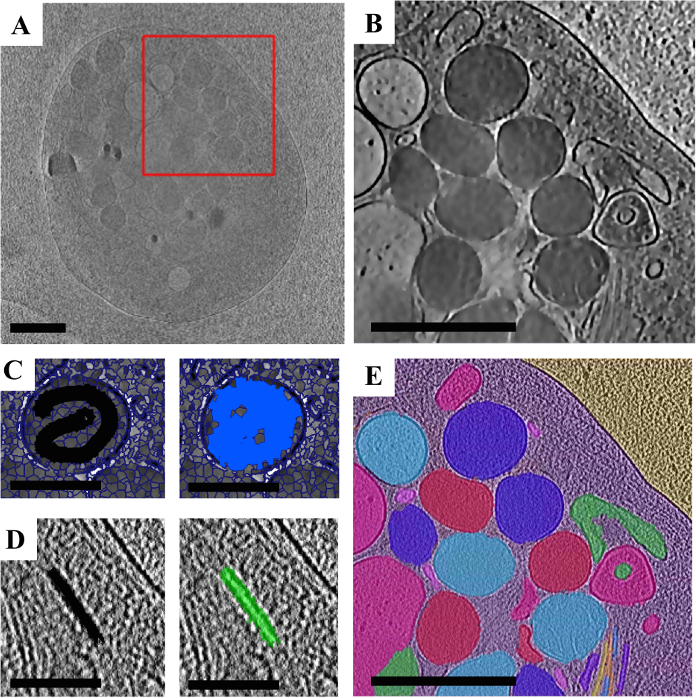

**Figure 4. Processing of a cryoET Dataset using a Super-region Segmentation Strategy. A.** A single 2D slice of the raw data. **B.** A region of interest (red box in **A**) with a layered filter set applied to accentuate the boundaries of the organelles. **C.** Example of annotating an organelle using super-regions. A single organelle is shown with supervoxels overlaid with the manual user annotation displayed in black (left) and the supervoxels chosen with that annotation shown in blue (right). **D.** Example of annotating a microtubule using super-regions. A single region of microtubule is shown with the manual user annotation displayed in black (left) and the supervoxels chosen with that annotation shown in green (right). **E.** The final segmentation featuring the platelet segmented from the background using model training (see Table 1 for details), and various organelles and microtubules segmented using a super-region segmentation strategy. Colors do not indicate specific organelle types as they are shown here before classification. Scale bars in A, B, and E are 1 μm and in C and D are 0.5 μm. Please click here to view a larger version of this figure.

**Figure:**
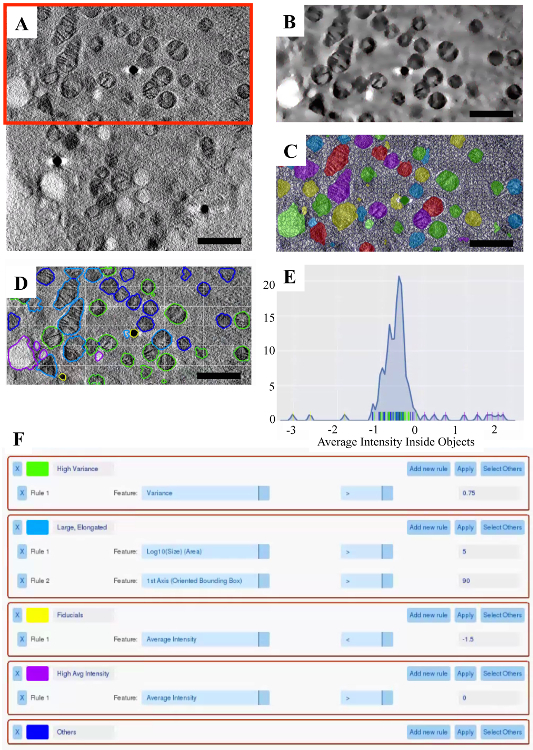

**Figure 5. Analysis of a cryoSXT Dataset using the Label Splitter Tool. A.** A single 2D slice of the raw data. **B.** A region of interest (red box in A) with a total variation filter applied to accentuate the organelles. **C.** The final segmentation with supervoxels overlaid. **D.** The visualization portion of the label splitter with the organelles classified using the rules displayed in **F**. **E.** The plot portion of the label splitter displaying the average intensity inside each object, with the rules displayed in** F** applied. Each vertical line along the x-axis represents a single object and is color-coded to match the class it has been assigned to. **F.** Example classification rules to separate various objects based on their inherent properties. Scale bars are 1 μm. Please click here to view a larger version of this figure.

**Table:** 

**Name / Dataset**	**Source**	**P1**	**P2**	**P3**	**P4**
**Gaussian Filter**		**Sigma**			
**Range / Default**	-	[0.5, 10] / 1			
(G1) cryoET	Raw data	1			
(G2) cryoET	Raw data	2			
**Total Variation**		**Lambda**	**Spacing**	**# Iter**	**Clamp**
**Range / Default**	-	[0.1, 30] / 10	[0.1, 10] / 1	[50, 500] / 100	-
(TV1) microCT	Raw data	10	1	100	(1, -)
(TV2) cryoET	G1	7	1	200	-
(TV3) cryoET	G2	10	1	100	-
(TV4) cryoSXR	Raw data	7	1	100	-
**Thresholding**		**Vmin**	**Vmax**		
**Range / Default**					
(TH1) cryoET	TV3	0	-		
**Gaussian Centering**		**Sigma**			
**Range / Default**	-	[0.5, 10] / 2			
(GC1) microCT	TV1	2			
**Gaussian Normalization**		**Sigma**			
**Range / Default**	-	[0.5, 10] /2			
(GN1) microCT	TV1	2			
**Laplacian of Gaussian**		**Sigma**	**Thresh**	**Response**	
**Range / Default**	-	[0.5, 10] / 2	[Yes/No] / No	[Bright/Dark] / Bright	
(LG1) microCT	TV1	2	No	Bright	
**Difference of Gaussians**		**Sigma Init**	**Sigma Ratio**		
**Range / Default**	-	[0.5, 10] / 2	[1.1, 3] / 1.6		
(DG1) microCT	TV1	2	1.6		
(DG2) cryoET	TV3	2	1.6		
**Det. Structure Tensor**		**Sigma1**	**Sigma Area**		
**Range / Default**	-	[0.5, 10] / 2	[0.5, 10] / 2		
(ST1) cryoET	TV3	2	2		
**Supervoxels**		**Shape**	**Spacing**	**Compactness**	
**Range / Default**	-	[1, 10] / 10	[0.1, 5] / 1	[1, 200] / 20	
(SV1) microCT	TV1	(10, 10, 10)	(1, 1, 1)	30	
(SV2) cryoET	TV3	(10, 10, 10)	(1, 1, 1)	50	
(SV3) cryoET	TV2	(3, 5, 5)	(1, 1, 1)	50	
(SV4) cryoSXT	TV4	(10, 10, 10)	(1, 1, 1)	30	
**Megavoxels**		**Lambda**	**# Bins**	**Gamma**	
**Range / Default**	-	[0.01, 1] / 0.1	[10, 200] / 20	None, auto or [0, 1] / None
(MV1) cryoET	SV2	0.1	50	auto	
TV1
(MV2) cryoSXT	SV4	0.4	50	None	
TV4
**Model training**		**Region**	**Classifier**	**Refinement**	
**Available / Default**		[voxel / supervoxel]	[Ensembles, SVM, Online Linear Models] /	[None, Potts, Appearance] / Appearance	
Ensemble - RF
microCT	TV3	SV1	Random Forest:	Appearance	
TH1		
GC1	- # Tree:	- Lambda:
GN1	[10, 100] / 100	[1, 500] / 10
LG1	100	50
DG1		
cryoET	TV2 DG2 ST1	SV2	Random Forest:	Appearance	
	
- # Tree:	- Lambda:
[10, 100] / 100	[1, 500] / 10
100	50
**Annotation Refinement**		**Radius**			
**Range / Default**		[1, 20] / 1			
**microCT**					
Opening	5
Fill-Holes	1
Dilation	2
**cryoET**					
Opening	3
Fill-Holes	1
Dilation	2
**cryoSXT**					
Opening	3
Fill-Holes	1
Dilation	2

**Table 1. Optimized Parameters used to Process Each of the Three Datasets (microCT, cryoET, and cryoSXT).** For each parameter, a general purpose range and default are given. In many cases, filtered data is used as a source for downstream processing. In these cases, an abbreviation is used to denote the new source dataset. For example, G1 (the Gaussian filtered cryoET raw data) was used as input during a total variation filter to create TV2. Information is only presented for aspects of the Workbench that were used to process each dataset. For example, Model Training was not used during processing of the cryoSXT dataset presented here, therefore no parameters are given for this.

## Discussion

SuRVoS Workbench differs from other segmentation programs in that optimization of parameters is a necessary and important step prior to beginning the actual segmentation. In some manual or semi-manual segmentation programs, the user begins segmenting within moments of opening a new project. With SuRVoS, because large amounts of the volume will be segmented with very little user input and boundaries are delineated by the program, optimizing the parameters is critical to a successful segmentation. Specifically, feature channels and super-region building are two areas where attention should be paid.

### Feature channels and model training

In addition to the raw data, SuRVoS allows the user to create additional datasets or channels derived from an existing dataset. These channels can be created using a selection of computational methodologies or feature extractors. Each of the data representations are available in parallel, and can be individually displayed to assess outcomes of feature or filter application. Because of these characteristics, they are referred to as feature channels in SuRVoS. There are many feature channel options provided within SuRVoS. For information on the options and parameters used here, see **Table 1**, for a full list and description of available feature channels visit https://diamondlightsource.github.io/SuRVoS/ 2. First, noisy datasets will benefit from denoising with either the Gaussian or total variation filter. It is recommended that further feature channel and supervoxel/megavoxel computations be performed using one of these denoised datasets as the data source. Generally, the total variation denoised dataset is used as the source data for feature channel and supervoxel/megavoxel computations. It is suggested to run with the default values first, assess the result in 3D and finally, iteratively optimize the parameters for the dataset. Additionally, feature channels can be built up into "filter sets" to specifically isolate aspects of the dataset, and these can then be used as data sources to create supervoxels and megavoxels. While this strategy is highly dataset dependent, it can be beneficial.

Feature channels are also used as sources to train the classifier in model training. When deciding on which feature channels to use, it is recommended that a few robust feature channels (*e.g.*, from blob detection, texture and structure, or robust features categories) are used when working with a small amount of annotations to train the classifier. When working with a large amount of training data, it is recommended to use more feature channels overall, from any of the categories as long as they provide varied information to the classifier (*e.g.*, add to the above list feature channels from local features and Gaussian features categories).

There are three main parts to model training: providing input data sources that describe the data, using these inputs to train a classifier, and finally refining the output predictions. Generally, smaller regions of the data will require more user annotations to accurately train the classifier, while larger regions of the data will require fewer user annotations. Model training first without selecting a refinement can be used to find the best predictions. Then include the refinement and optimize the lambda parameter as needed to fix issues with the predictions such as holes or jagged edges.

### Supervoxels and megavoxels

Supervoxels are clusters of multiple nearby, similar voxels^38^,^39^. Supervoxels begin as a standard 3D grid overlaid on the data that is then iteratively deformed in order to adhere to the underlying boundaries, and thus better represent the data. Supervoxel creation and deformation is controlled by four user inputs: data source, superpixel shape, spacing, and compactness. The data source provides the data inputs that are queried during supervoxel creation. Any source can be used including filtered data sources. The superpixel shape parameters determine the starting 3D grid and the approximate desired shape of the resulting supervoxels. Changing these parameters can increase or decrease the size of the supervoxels before deformation. The spacing parameters define the importance of boundaries in each direction. Changing these parameters can emphasize boundaries in one or two directions at the expense of the other(s), meaning the resulting supervoxels will deform to better follow data boundaries in the given direction(s). The last parameter, compactness, controls how much the supervoxels can deform. A low compactness number allows the supervoxels to deform more. These parameters should be optimized to provide supervoxels that represent the boundaries of the data of interest. Note: Currently, supervoxel shape parameters must equal 1024 or less when multiplied together.

In some ways, supervoxel parameters can compensate for each other, meaning there is no one "right answer" when deciding on parameters. For example, a large starting grid (*e.g. *superpixel shape: 10 x 10 x 10) and a low compactness number (ex. 20) may give supervoxels with similar boundary adherence as compared to a small starting grid (*e.g.* superpixel shape 5 x 5 x 5) and a higher compactness number (*e.g.* 50). Because there are more, smaller supervoxels in the second scenario, they don't have to deform as much to represent boundaries. Both sets of parameters could be appropriate for segmentation of the dataset.

The biggest consideration when choosing supervoxel parameters is how well the supervoxels represent the data. Displaying the supervoxels alone, without data underneath them, as in Figure 2D, is a good way to assess supervoxel parameters. When displayed this way, the edges and outlines of shapes found in the data should still be visible in the supervoxels.

Megavoxels are conglomerates of multiple neighboring, similar supervoxels^38^,^39^. They are again controlled by four user inputs: data source, lambda, numbins, and gamma. As with supervoxels, the data source provides the data inputs that are queried during megavoxel creation. Both lambda and numbins impact the size and boundary adherence of the megavoxels. As the megavoxels grow larger (high lambda, low numbins), their boundary adherence decreases. The converse is also true, boundary adherence will increase with smaller megavoxels (low lambda, high numbins), however as the megavoxel size decreases, so does their usefulness in segmenting large amounts of voxels quickly. The optional gamma parameter controls the smoothness factor versus the cost of merging two supervoxels together. Small values of gamma can enhance the similarity between two supervoxels, at the cost of having fewer megavoxels overall.

As with supervoxels, the biggest consideration when choosing and optimizing megavoxel parameters is how well the megavoxels represent the data. Displaying the megavoxels alone as described for supervoxels can again be used to assess parameters. However, because megavoxels will generally be much larger and are three-dimensional, using the annotation tools to choose single megavoxels to ensure the boundary between regions of interest is tight is also recommended.

### Annotation strategy

Two general annotation strategies have been described: a model training approach is useful for separating large regions of a dataset, while a super-region segmentation approach is useful for smaller, more diverse features such as individual organelles. Annotations can be organized in a hierarchical fashion so that it is possible to annotate large regions first, then subdivide them into more specific regions using a parent-child relationship. The parent label for a label can be assigned by clicking on the area to the right of the label color selection and choosing an appropriate parent label from a previous level. In practice, most datasets use both the model training and super-region segmentation strategies to segment specific regions/features of interest.

In the model training example here, a few training inputs (in the form of manual user supervoxel-based annotations) were used on three equally spaced slices of the data. In this way, the model training aspect of SuRVoS vastly increases the speed with which segmentation is possible especially when working with large, differentiated regions such as the divide between the regions in the goosegrass fruiting body as highlighted in Figure 3.

When model training, if the predictions cannot be seen, it may be necessary to go to the Visualization tab and make sure that the Predictions layer is turned on and set to an appropriate amount of transparency. Also, a confidence of 0 will assign every unlabeled supervoxel to a label, based on whatever the closest match is. Confidence of 100 will only assign a label if only one category of label has any proportional match. Everything in between is a tradeoff of these two extremes. When selecting a confidence level it is suggested to check a few slices to visually inspect that there are no incorrectly predicted voxels prior to saving the prediction to a label.

A good strategy for annotating using super-regions is to use the magnification tool to zoom in on the data, annotate a few organelles at once on one slice, using a "quick, messy" approach first (Figure 4C). Next, move up or down a few slices in Z and repeat this process. Because supervoxels are three-dimensional, many of the faults of the "messy" approach are fixed by annotations made in the above or below slices. In this way, segmentation is sped up and the boundaries are provided by the supervoxels rather than manually.

To clean up a label, standard segmentation refinement options have been provided. Dilation causes the selected segmentation label to grow by the given radius, erosion causes it to shrink. Opening and closing are the application of first erosion and then dilation, or vice-versa, respectively. And fill holes does exactly that. The order of these operations does matter. Generally, performing fill holes, then opening, then dilation works well. Each refinement method can be applied on a single slice ("this slice"), on all slices in 2D ("All slices (2D)") or in 3D ("Whole volume (3D)"). All slices (2D) is recommended.

### Significance and future directions

Efficient and accurate segmentation is the next bottleneck in processing of 3D datasets, especially with the routine automated collection of terabytes of image data during long-run sessions. SuRVoS Workbench can speed the segmentation process by a factor of 5 as compared to manual segmentation. Also, because the boundaries are delineated by supervoxels, the variability of the resultant segmentations should improve. In future, we hope to explore ways of using the segmentation of a representative 3D region of interest as training data to apply to the rest of the volume, or even a separate volume, with high confidence. This advance would further decrease the amount of user time and input necessary to segment even complex biological volumes, helping to alleviate the image processing and segmentation bottleneck. This, in turn, will allow the quantitative comparison of biological data in various states (e.g. non-disease, disease, treated) with robust experimental numbers.

## Disclosures

The authors declare they have no competing financial interests.
